# Improving the Quality of Pediatric Basic Life Support Cardiopulmonary Resuscitation With a Novel Method: The Maryland Hiccup

**DOI:** 10.7759/cureus.78783

**Published:** 2025-02-09

**Authors:** Jennifer F Anders, Camille Anderson, Cynthia Wright-Johnson, Karen J O'Connell

**Affiliations:** 1 Pediatric Emergency Medicine, Johns Hopkins University School of Medicine, Baltimore, USA; 2 Pediatric Critical Care Medicine, Johns Hopkins University School of Medicine, Baltimore, USA; 3 Emergency Medical Services (EMS) for Children, Maryland Institute of Emergency Medical Services Systems, Baltimore, USA; 4 Pediatric Emergency Medicine, George Washington University School of Medicine and Health Sciences, Washington, DC, USA

**Keywords:** basic life support, cardiac arrest, cardiopulmonary resuscitation, cpr, emergency medical services, pediatric out-of-hospital cardiac arrest, prehospital

## Abstract

Prehospital high-performance cardiopulmonary resuscitation (CPR) has demonstrated remarkable improvements in the survival of adult patients after out-of-hospital cardiac arrest (OHCA). With a goal to improve pediatric survival rates, Maryland Emergency Medical Services (EMS) for Children created a pediatric-specific high-performance CPR protocol to align with the existing state protocol for adult CPR. While prehospital CPR for adults has moved toward continuous compressions, prehospital CPR for children without an advanced airway continues to adhere to American Heart Association (AHA)/International Liaison Committee on Resuscitation (ILCOR) guidance for two ventilations for every 15 compressions. The Maryland Hiccup (MH) is a novel approach to pediatric CPR. The method combines the goal of continuous compressions with AHA/ILCOR guidance for a 15:2 compression-to-ventilation ratio. In contrast to the two- to four-second pause after compression 15 of the AHA/ILCOR style, the "hiccup" of the MH method describes two brief pauses for ventilation during the upstrokes of compressions 14 and 15.

We compare basic CPR quality metrics for two-rescuer high-performance CPR using the standard AHA/ILCOR 15:2 style compared to the MH style. We enrolled 38 Maryland EMS clinicians in two-person teams to perform simulated CPR on a pediatric manikin. We recorded compression and ventilation data for 76 two-minute cycles of high-performance CPR.

Compression fraction was significantly improved using the MH when compared to the standard AHA style for high-performance CPR (median 98% vs 80%, p<0.001). When compressions per minute (CPM) were compared by 30-second epochs, 80% of epochs were out of range (<100 CPM or >120 CPM) using the AHA/ILCOR style, while only 32% of epochs were out of range for MH style. No significant differences were found between the two CPR styles in ventilation volume or compression depth.

The MH is a novel method for pediatric basic life support two-provider CPR which improves CPR quality metrics among EMS clinicians regardless of their prior experience. The use of this method in simulation allows near-continuous compressions without the placement of an advanced airway. Future research is needed to explore whether the CPR quality improvements translate to improved patient outcomes in real-world use.

## Introduction

Over the past two decades, survival after pediatric in-hospital cardiac arrest (IHCA) has improved significantly (from approximately 19% in 2000 to 38% in 2018) [1]. Over the same time, survival after out-of-hospital cardiac arrest (OHCA) for children has improved modestly (approximately 5 to 8%) [2,3]. Much improvement in IHCA has been credited to improvement in cardiopulmonary resuscitation (CPR) quality, including compliance with the current American Heart Association (AHA)/International Liaison Committee on Resuscitation (ILCOR) compression depth and rate guidelines [4-6]. It has also been recognized that while endotracheal intubation (ETI) has a negative impact on survival from both in- and out-of-hospital cardiac arrest, adequate ventilation is a key factor for pediatric patient survival [7,8].

Efforts to improve survival after OHCA are focused on prehospital basic life support (BLS) skills, such as CPR. Prehospital CPR for adult patients has seen widespread quality improvements, including the development of high-performance CPR (also known as pit crew CPR or team-focused CPR) which mimics the guidelines for in-hospital high-quality CPR, and emphasizes early defibrillation, rate and volume-controlled ventilation, quality chest compression metrics, and minimal compression interruptions [9-11]. Acknowledging the critical importance of early and high-quality chest compressions, adult CPR guidelines have been modified to promote compression-only CPR directed by dispatchers or performed by bystanders unwilling or untrained to perform rescue breaths [12-15]. To address the negative impact of ETI on patient outcomes, AHA/ILCOR guidance for adults with OHCA supports either a 30-compressions to two-ventilations ratio or continuous compressions with bag-valve-mask (BVM) ventilations by EMS [14].

In contrast to adult cardiac arrest, pediatric cardiac arrest has a high likelihood of respiratory failure as the causative etiology [16]. Studies comparing compression-only versus conventional CPR in children have shown better outcomes when rescue breaths are performed in conjunction with chest compressions for most scenarios [17-19]. While the AHA Pediatric Advanced Life Support (PALS) subcommittee has created an allowance for compression-only CPR by lay people as marginally better than no CPR, it has been steadfast in recommending the 15-compressions to two-ventilations ratio for CPR by healthcare professionals for infants and children when an advanced airway (i.e., endotracheal tube or supraglottic airway) is not in place [20]. The pause in compressions for ventilation is intended to facilitate thoracic expansion to allow for adequate tidal volumes. Additional confusion regarding best practices for pediatric OHCA arises from the knowledge that intubation is a negative predictor of good outcomes, and that endotracheal intubation attempts have been associated with prolonged compression pauses [21,22]. Minimizing pauses in chest compressions has been identified as a critically important component of high-quality CPR [19] and efforts to improve survival from OHCA in children may benefit from a focus on this quality metric. These differences in guidelines between adult and pediatric CPR priorities create contradictory instructions and confusion for prehospital clinicians.

As Maryland Emergency Medical Services (EMS) adopted a high-performance CPR protocol for children, EMS clinicians and medical directors sought to harmonize existing adult CPR guidelines recommending continuous compressions with the pediatric AHA guidelines for a 15:2 compression-to-ventilation ratio. We created a novel CPR style referred to as the Maryland Hiccup (MH). The objective of this work was to test the quality of CPR delivered by EMS clinicians using the standard AHA style compared to the novel MH style.

## Materials and methods

In MH high-performance CPR, the 15:2 compression-to-ventilation ratio is preserved, with one ventilation given on the upstroke of compression 14 and a second on the upstroke of compression 15 (Video 1).

**Video 1:** Demonstration of MH CPR with 15:2 compression:ventilation ratio. In the MH method of CPR, the ILCOR guideline ratio of two ventilations for every 15 compressions is preserved. In each 15:2 cycle, the first ventilation is delivered on the upstroke of compression 14, and the second ventilation is delivered on the upstroke of compression 15. The compressor maintains team synchronization by verbalizing the compressions and the pause for ventilation. MH, Maryland Hiccup; CPR, cardiopulmonary resuscitation; ILCOR, International Liaison Committee on Resuscitation.

**Figure 1:**
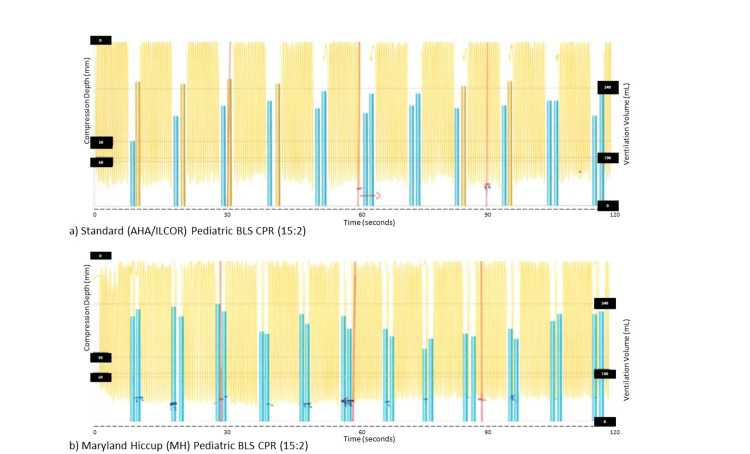
Recorded compressions and ventilations for sample two-minute cycles of pediatric two-person CPR using 15:2 compression to ventilation ratio in (a) standard AHA/ILCOR style CPR and (b) MH CPR. Chest compression (thin yellow lines) is displayed as millimeters of compression depth from the top down. Ventilations (thick blue and yellow bars) are displayed as milliliters of ventilated volume from the bottom up.  The blue bars represent breaths within the goal volume and the yellow bars represent breaths that are under or over the goal volume. The x-axis displays the passage of time with marks at each 30-second interval. AHA, American Heart Association; ILCOR, International Liaison Committee on Resuscitation; MH, Maryland Hiccup; CPR, cardiopulmonary resuscitation; BLS, basic life support.

Figure 1 illustrates the pattern of compressions and ventilations with the standard AHA/ILCOR style (Figure 1a) and the MH style (Figure 1b). In practice, the compressor allows a brief pause or “hiccup” on the upstroke but otherwise performs continuous compressions. (Compressors are instructed to verbalize the compression number and cue their partner to ventilate. For example, “…12, 13, 14 - breathe, 15 - breathe, 1, 2…”.) The theoretical maximum chest compression factor (CCF) with this method is >98%. 

We conducted a prospective, randomized crossover study of CPR quality in simulated pediatric OHCA comparing the standard AHA style 15:2 compression to ventilation ratio with the MH style.

In preliminary studies where pediatric high-performance CPR with the hiccup method was taught to experienced CPR providers, we saw a mean improvement of CPR quality score (Laerdal SimPad®, Laerdal Medical, Stavanger, Norway) by more than 30 points (52 to 87) with a standard deviation of 20 points. We calculated a sample size of 16 participants per clinician level (16 BLS and 16 ALS) with the goal of detecting at least a 20-point difference in CPR score between the two methods, with 80% power and 95% validity.

We recruited a convenience sample of EMS clinicians in two-person teams to perform simulated high-performance CPR on the Laerdal SimJunior® (Laerdal Medical, Stavanger, Norway) manikin representing a six-year-old boy. Volunteers qualified for inclusion if they were EMS clinicians certified at the emergency medical technician (EMT) level or higher, currently affiliated with a Maryland EMS operation program, and currently certified in BLS CPR. Participants received no compensation for this study. The study was approved by the Johns Hopkins Medicine Institutional Review Board (approval number 00255214, dated August 7, 2020) as human subjects research and conducted in accordance with international ethical standards for research conduct. All EMS clinician volunteers completed signed, informed consent prior to participation.

Participants received no study-specific training in CPR but were provided with a one-page card to review while waiting and were permitted to observe other research participants performing CPR. Each team performed two two-minute cycles of high-performance CPR in one style, then crossed over to perform two two-minute cycles in the other style. Teams were randomized to the order they performed the two CPR types and individuals were randomized to the order in which they took the ventilator or compressor role.

Data was captured using the Laerdal SimJunior® manikin with SimPad® and SkillReporter® software (Laerdal Medical, Stavanger, Norway). Each two-minute session was analyzed as a whole using the SimPad® calculated metrics report for CPR quality score, compression rate, compression fraction, compression depth, and ventilation volume. In addition, the comprehensive recording of each simulation session (Figure 1) was analyzed to count the actual number of compressions delivered and to measure the actual total pause duration for ventilation during each 30-second epoch of CPR. Pause for ventilation was defined as the time between the completion of the upstroke prior to ventilation and the start of the downstroke of the following compression; total pauses were summed for each 30-second epoch.

The primary comparison of interest was the difference in CPR quality between MH and AHA/ILCOR CPR. CPR quality metrics included the four core metrics of compression rate, compression depth, compression fraction, and ventilation volume, and additionally, the overall quality score reported by Laerdal SimPad® and SkillReporter® software. CPR quality score is reported from 0 to 100% and is derived by a complex calculation that is proprietary. Medians for each metric were compared by Chi-square test and reported with interquartile ranges (IQRs). Comparisons were repeated in subgroups divided by level of certification (ALS vs BLS) and prehospital experience (three or fewer years versus greater than three years). In addition, the number of epochs with compression rates in or out of the desired range was compared with the Student's t-test.

## Results

We enrolled 38 EMS clinicians affiliated with one of four geographically diverse Maryland EMS agencies; 22 (58%) clinicians credentialed at the BLS level and 16 (42%) credentialed at the ALS level.

Clinician years of experience in EMS ranged from one to 35 years (median of 11.5 years with IQR 4-21 years) and self-report estimated medians of seven CPR episodes in the prior year but less than one pediatric CPR episode in the prior year. Eighty-nine percent reported prior training in pediatric high-performance CPR. Clinicians were defined as “low experience” if they had three or fewer years of EMS work experience and “high experience” if they had greater than three years of EMS experience.

The 19 teams performed a total of 76 two-minute sessions of CPR which we divided into 304 30-second epochs. The manikin’s ventilation sensor did not function for 10 of the simulated CPR sessions (five in the AHA group and five in the MH group); thus, only 66 two-minute cycles (264 30-second epochs) included measurement of ventilation volumes.

CPR quality metrics

CPR quality score reported by the Laerdal SimPad® was higher for the AHA/ILCOR method than the MH method (88 vs. 81; p=0.04) but not statistically significant in the EMS clinician subgroups by credential level or experience. Quality score was considered only for those sessions where ventilation data was recorded (n=66) (Table 1).

**Table 1:** Comparison of CPR quality score for CPR style and experience and EMS clinician credential level. CPR, cardiopulmonary resuscitation; EMS, emergency medical services; ALS, advanced life support (clinician certification level); BLS, basic life support (clinician certification level); AHA/ILCOF, American Heart Association/International Liaison Committee on Resuscitation CPR method; MH, Maryland Hiccup CPR method.

CPR quality score (n=66)	AHA/ILCOR (n=33)	MH (n=33)	p-value
Overall	88 (72-94)	81 (72-89.5)	0.04
ALS compressor	93 (86-94)	83 (73-90)	0.19
BLS compressor	86 (69-91)	80 (69-87)	0.50
High-experience compressor	89 (77-93)	83 (73-89)	0.16
Low-experience compressor	79 (68-97)	69 (61-92)	0.81

Using the basic data supplied by the Laerdal SimPad® CPR assessment, we found a significant improvement in the chest compression fraction when using the MH method compared to the AHA method (100% vs 78% respectively; Table 2). 

**Table 2:** Comparison of compression metrics (chest compression fraction, rate, actual compressions delivered per minute, and depth) for AHA and MH CPR Styles for all EMS clinicians and subsets of compressors by certification and experience levels. CPR, cardiopulmonary resuscitation; EMS, emergency medical services; IQR, interquartile range; ALS, advanced life support (clinician certification level); BLS, basic life support (clinician certification level); AHA, American Heart Association CPR method; MH, Maryland Hiccup CPR method.

Chest compression metrics for 2 min cycles of simulated CPR median with (IQR)	AHA (N=38)	MH (N=38)	p-value
Chest compression fraction	% (IQR)	% (IQR)	
Overall	78 (74-84)	100 (98-100)	<0.001
ALS compressor	76 (73-79)	100 (99-100)	<0.001
BLS compressor	82 (76-90)	100 (97-100)	<0.001
High-experience compressor	78 (74-84)	100 (98-100)	<0.001
Low-experience compressor	76 (74-82)	100 (98-100)	<0.001
Chest compression rate (machine calculated)	median (IQR)	median (IQR)	
Overall	121 (115-126)	117 (115-121)	0.56
ALS compressor	119 (116-126)	117 (115-121)	0.87
BLS compressor	121 (114-123)	119 (113-122)	0.55
High-experience compressor	122 (118-126)	118 (114-121)	0.36
Low-experience compressor	113 (110-121)	116 (113-122)	0.64
Chest compressions delivered per minute	median (IQR)	median (IQR)	
Overall	92 (84-100)	104 (98-110)	<0.001
ALS compressor	92 (84-100)	106 (100-112)	<0.001
BLS compressor	92 (83-98)	102 (97-108)	<0.001
High-experience compressor median (IQR)	92 (86-100)	104 (98-111)	<0.001
Low-experience compressor median (IQR)	88 (82-92)	102 (97-108)	<0.001
Compression depth in mm	median (IQR)	median (IQR)	
Overall	62 (56-68)	60 (54-64)	0.20
ALS compressor	63 (60-68)	60 (55-64)	0.36
BLS compressor	62 (54-65)	60 (53-64)	0.76
High-experience compressor	62 (56-68)	60 (54-64)	0.36
Low-experience compressor	63 (56-68)	60 (57-64)	0.64

**Figure 2:**
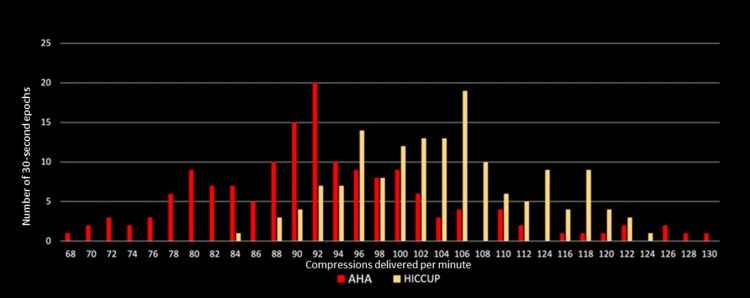
Compressions delivered for each 30-second epoch, expressed in compressions per minute (CPM). Red bars indicate number of epochs with each CPM using standard AHA 15:2 method (n=152) and yellow bars indicate number of epochs with each CPM using the MH method (n=152). AHA, American Heart Association CPR method; HICCUP, Maryland Hiccup (MH) CPR method.

The chest compression rate measured by Laerdal SimJunior® manikin did not differ significantly between the two methods (119/min with MH vs 116/min with AHA method). No difference was found between subsets of clinicians based on credential level or experience (Table 2). In contrast, a significant difference was noted when individual chest compressions delivered to the manikin were measured for each two-minute cycle (Table 2). Using the same count of chest compressions delivered per minute for each 30-second epoch (n=304), 53% (161) of epochs had a rate less than 100 per minute, 44% (133) were between 100-120 per minute, and 3% (10) were at rates greater than 120 per minute (Figure 2). Fewer epochs were associated with compression rates outside the ILCOR goal of 100-120 compressions per minute when the MH method was used than with the standard AHA/ILCOR method (32% vs 80% of epochs respectively, p<0.001).

Chest compression depth did not differ significantly between the two methods and there were no differences seen in subsets of clinicians based on experience or credential level (Table 2).

Ventilation volume was available for 66 of the 76 sessions. Actual pause durations for ventilation were measured from the recording of each session and were significantly shorter using the MH technique compared to the standard AHA technique (Table 3). There was no difference in mean ventilation volume between the two groups (127mL with the AHA method vs 114mL with the MH method, p=0.19). No difference was seen in subsets of clinicians based on credential level or experience (Table 3).

**Table 3:** Comparison of ventilation pauses and ventilation volumes for AHA and MH CPR Styles for all EMS clinicians and subsets of ventilators by certification and experience levels. CPR, cardiopulmonary resuscitation; EMS, emergency medical services; IQR, interquartile range; ALS, advanced life support (clinician certification level); BLS, basic life support (clinician certification level); AHA, American Heart Association CPR method; MH, Maryland Hiccup CPR method.

Median seconds for ventilation pauses per 30-second epoch (IQR) (N=304)	AHA (n=152)	MH (n=152)	p-value
Overall	7.27 (5.91-8.64)	4.09 (0.45-5.91)	<0.001
ALS ventilator	7.62 (5.91-8.64)	4.01 (0.45-5.84)	<0.001
BLS ventilator	7.62 (5.91-8.64)	4.01 (0.45-5.84)	<0.001
High-experience ventilator	7.27 (5.91-8.77)	3.92 (0.23-5.91)	<0.001
Low-experience ventilator	7.27 (6.59-8.31)	4.32 (2.05-5.45)	<0.001
Median ventilation volume in mL (IQR) (N=66)			
Overall	133 (79-163)	116 (84-143)	0.54
ALS ventilator	134 (89-167)	110 (89-120)	0.35
BLS ventilator	116 (67-155)	118 (67-144)	0.75
High-experience ventilator	122 (118-126)	118 (114-121)	0.36
Low-experience ventilator	104 (73-147)	120 (84-155)	0.81

## Discussion

The MH method allowed near-continuous chest compressions while delivering ventilations at a 15:2 ratio. Synchronized ventilations delivered on the upstrokes of the 14th and 15th chest compressions significantly improved several CPR quality metrics in simulated pediatric CPR by EMS clinicians. The MH method mimics the Resuscitation Academy method for adult high-performance CPR, where chest compressions are continuous with ventilations interspersed on the upstrokes every six seconds, yet adheres to the 15:2 ratio recommended by AHA/ILCOR for pediatric CPR [11].

Early CPR performed by laypersons or first responders is recognized as a key to the chain of survival after out-of-hospital cardiac arrest. Layperson compression-only CPR has become standard for adult patients, and in many jurisdictions, EMS clinicians also focus on continuous compressions with interspersed ventilations. In contrast, for children with OHCA, CPR with rescue breathing ventilation has been shown to improve outcomes of CPR performed by both laypersons and healthcare providers [19]. The differing standards between adult and pediatric CPR add challenge and it is recognized that pediatric resuscitations are both more error-prone and create more cognitive stress for clinicians [23]. The MH method aligns pediatric and adult high-performance CPR training to increase usability and improve CPR quality during these high-stress events.

EMS clinicians are healthcare providers with extensive training and experience in the provision of high-performance CPR for patients of all ages. While adult CPR is frequently performed by most clinicians, pediatric cardiac arrest is much less commonly encountered and pediatric CPR is less frequently performed. As a result, teaching high-performance CPR to EMS focuses primarily on adult resuscitation and emphasizes the provision of continuous compressions. The time available for teaching pediatric high-performance CPR to EMS is precious, with limited teaching opportunities and many competing priorities. Therefore, the educational method should align as closely as possible with adult CPR. The MH style of pediatric BLS CPR does this while stressing the importance of providing rescue breathing during nearly continuous chest compressions.

Pediatric high-performance CPR was adopted as a Maryland statewide protocol in 2018 [24]. This protocol endorses key principles of high-performance CPR and adheres to current AHA PALS recommendations of 15:2 compressions to ventilation ratio. The MH method was introduced during statewide roll-out training for pediatric high-performance CPR. We conducted 1.5-hour workshop training sessions and witnessed the rapid uptake of the MH method by EMS clinicians. Training hundreds of clinicians in the roll-out, we measured dramatic improvement in CPR quality scores for participants (overall mean of 53 before to 87 after, p<0.001). Our experience with the rapid uptake of the MH method in EMS training and its strict adherence to the international (AHA/ILCOR) recommendation for pediatric BLS CPR both suggest that the method holds great potential for adoption across EMS systems.

While the MH method presents an elegant solution to improve compression fraction while delivering adequate ventilation to children in cardiac arrest, there are challenges to its acceptance. The “hiccup” requires more complex teamwork between clinicians organizing compressions and ventilations than the standard AHA/ILCOR method. Adoption of a new method like the MH requires investment in training and regular pediatric CPR drills to reinforce the team choreography.

Of note, the CPR quality score did not improve with the MH in this study, in contrast to our prior experience with high-performance CPR teaching. In those sessions, we taught the MH method as part of a comprehensive workshop focused on principles of high-performance CPR including rapid cycle practice and teamwork. Furthermore, in this study, the CPR quality score was lower using the MH, while multiple individual CPR quality metrics (compression fraction, rate) were significantly higher with the MH and none were significantly worse (ventilation volume, compression depth). Further study is needed to know if this is a limitation of the calculated CPR quality score which is designed to assess compliance with the expected 15:2 method or if there is an unintended negative impact of the MH.

Our study has several limitations to recognize. First, the manikin is an artificial system and assesses only CPR quality metrics. The simulation setting is a “best case” scenario with minimization of the stressors and competing priorities that normally exist at a pediatric cardiac arrest scene. Second, the gold standard for EMS high-performance CPR quality is unclear. National organizations providing EMS high-performance CPR training and guidelines for resuscitation suggest that a CCF should be as high as possible, but no significant improvement in outcome was seen with continuous vs. interrupted compressions in the Resuscitation Outcomes Consortium study [11,25]. The current AHA guidelines reinforce the importance of delivery of ventilations to children in cardiac arrest, and a newer study of children in the inpatient critical care setting showed improved survival and outcomes when ventilations were supported, in particular at higher rates than currently recommended [26]. Finally, no return of spontaneous circulation or survival was measurable on the manikins so we can only hypothesize that this high-performance CPR style may lead to better outcomes but this requires study in animal models or human patients.

## Conclusions

This study of CPR styles in simulated pediatric OHCA resuscitation demonstrated improved chest compression fraction, more chest compressions delivered per minute, and total pause for ventilation significantly decreased using the MH method compared to the AHA/ILCOR method of pediatric BLS CPR. At the same time, no degradation was seen in compression depth or ventilation volume using the MH compared to the AHA/ILCOR method. 

Pediatric OHCA is a low-frequency, high-stakes event for EMS clinicians. Pediatric resuscitation by EMS clinicians has a higher cognitive load and a higher rate of error when compared to adult resuscitation. Training and maintenance of pediatric CPR skills is a challenge for EMS educators and EMS operational systems. The MH method for pediatric BLS CPR presents a viable alternative to the existing AHA standard method. Further evaluation of the MH method in animal models or during actual resuscitations of pediatric patients is warranted.
